# Phosphodiesterase type 5 inhibition may reduce diastolic function in women with ischemia but no obstructive coronary artery disease

**DOI:** 10.1186/s13256-017-1307-2

**Published:** 2017-05-22

**Authors:** Michael D. Nelson, Puja K. Mehta, Janet Wei, Behzad Sharif, Louise E. J. Thomson, Daniel Berman, Debiao Li, C. Noel Bairey Merz

**Affiliations:** 1 Barbra Streisand Women’s Heart Center, Cedars-Sinai Heart Institute, 127 S. San Vicente Blvd, Suite A3600, Los Angeles, CA 90048 USA; 2 Biomedical Imaging Research Institute, Cedars-Sinai Medical Center, Los Angeles, CA USA; 3 Applied Physiology and Advanced Imaging Laboratory, University of Texas at Arlington, Arlington, TX USA; 4 S. Mark Taper Foundation Imaging Center, Cedars-Sinai Medical Center, Los Angeles, CA USA

**Keywords:** Women’s ischemic syndrome, Coronary microvascular dysfunction, Diastolic dysfunction, Myocardial perfusion reserve index, Phosphodiesterase type 5 inhibition

## Abstract

**Background:**

Ischemia, in the absence of obstructive coronary artery disease, is prevalent in women, and associated with increased risk for major cardiovascular events. Coronary microvascular dysfunction is prevalent in these patients, and associated with impaired diastolic function. Despite our general understanding, however, optimal treatment of this cohort remains elusive.

**Methods:**

To address this knowledge gap, we performed an open-label treatment trial to assess whether phosphodiesterase type 5 inhibition improves coronary microvascular perfusion and diastolic function in women with signs and symptoms of ischemia but no evidence of obstructive coronary artery disease. Left ventricular morphology and function, along with myocardial perfusion reserve index, were assessed by contrast-enhanced cardiac magnetic resonance imaging.

**Results:**

A total of five women enrolled of which four completed the trial, while one was withdrawn by the investigators after developing dyspnea 1 week after treatment. Her symptoms resolved after cessation of the study medication. In contrast to our hypothesis, phosphodiesterase type 5 inhibition reduced the rate of circumferential strain in diastole in all four women who completed the trial (that is, diastolic dysfunction). This impairment could not be explained by changes in heart rate, contractility, blood pressure, or preload, and was not associated with a change in myocardial perfusion reserve index. Frequency of angina also tended to increase with treatment, with the greatest increase occurring in the patient with the greatest impairment in diastolic strain.

**Conclusions:**

Taken together, these data question the efficacy of phosphodiesterase type 5 inhibition to treat women with ischemic heart disease, and highlight the need for further investigation.

## Background

Ischemia, in the absence of obstructive coronary artery disease (CAD), is prevalent in women, and associated with increased risk for major cardiovascular events, including myocardial infarction, stroke, heart failure, and sudden cardiac death [1]. Sex-specific research initiatives, including the Women’s Ischemia Syndrome Evaluation (WISE) study sponsored by the National Heart, Lung, and Blood Institute (NHLBI), have established coronary microvascular dysfunction (CMD) and diastolic dysfunction as important etiologic features of this disease [2, 3]. Despite these advancements, effective treatment remains elusive [4].

Phosphodiesterase (PDE) 5 is expressed in vascular smooth muscle cells and regulates vasorelaxation by catabolizing cyclic guanosine monophosphate (cGMP), the downstream target of nitric oxide. PDE5 is upregulated in stress states [5], and thus could contribute to the pathophysiology of ischemic heart disease in women. Accordingly, we sought to determine whether PDE5 inhibition improves CMD-related perfusion and diastolic function in women with signs and symptoms of ischemia but no obstructive CAD.

## Methods

Patients were recruited from the WISE-Coronary Vascular Dysfunction study (NCT00832702), which is a NHLBI-sponsored investigation designed to improve diagnostic testing and advance new hypotheses relative to the pathophysiology of ischemic heart disease in women. Women undergoing clinically ordered coronary angiography for suspected ischemia, but without obstructive CAD [6], were recruited. Exclusion criteria included: age <18 years, body mass index (BMI) ≥44 kg/m^2^, irregular heartbeat, renal failure, concurrent use of nitrates, alpha-adrenergic receptor blockers, or PDE inhibitors, as well as any contraindication to magnetic resonance imaging (MRI).

Patients were treated for 2 weeks in an open-label, non-randomized protocol with the PDE5 inhibitor tadalafil or sildenafil, as per the ordering physician’s instructions (Table 1). In all cases, the final capsule was ingested the night before the follow-up MRI.

**Table 1:** Patient characteristics, medical history, treatment history, and medication usage

	Patient #				
	1	2	3	4	5
Age, years	55	67	56	65	82
Body mass index, kg/m^2^	23	26	21	29	19
Heart rate, bpm	53	63	80	48	41
Systolic blood pressure, mmHg	126	96	153	112	100
Diastolic blood pressure, mmHg	53	53	81	59	53
LV end-diastolic pressure, mmHg	15	17	---	20	---
Medical history					
Hypertension	---	Y	Y	Y	Y
Dyslipidemia	Y	Y	---	Y	---
Diabetes mellitus	---	---	---	Y	---
Tobacco smoking	---	---	---	---	---
Myocardial infarction	---	---	---	Y	---
PDE5-inhibitor					
Drug	Tadalafil	Tadalafil	Tadalafil	Sildenafil	Sildenafil
Dose	5 mg	20 mg	5 mg	20 mg TID	10 mg TID
Frequency	Every 3 days	Every 3 days	Every 3 days	Daily	Daily
Concomitant medications					
Antiplatelet	Aspirin	Aspirin	Aspirin	Aspirin	Aspirin
Beta-blocker	Carvedilol	Atenolol	---	Carvedilol	Atenolol
Calcium channel blocker	---	---	Diltiazem	---	---
Statin	Pitavastatin	Rosuvastatin	---	Atorvastatin	---
Diuretic	---	HCTZ	---	---	HCTZ Furosemide
ACE-I/ARB	---	Ramipril	Losartan	Lisinopril	Losartan
Other	Levothyroxine	Amitriptyline	---	Metformin	Levothyroxine
Other	Prometrium (progesterone)	---	---	---	Dronedarone
Other	Estradiol	---	---	---	---

*ACE-I/ARB* angiotensin-converting enzyme inhibitor/angiotensin receptor blocker, *bpm* beats per minute, HCTZ hydrochlorothiazide, *LV* left ventricular, *PDE5* phosphodiesterase 5, *TID* three times a day, *Y*, Yes. Patient 5 was withdrawn by the investigators after developing dyspnea 1 week after treatment

Cardiac MRI was performed at baseline and after 2 weeks of treatment (Siemens 3 T Verio; Erlangen, Germany). Imaging included cines for morphological and functional analysis (four chamber), mid-ventricular short-axis tissue tagging for strain analysis (HARP, Diagnosoft; Durham, NC, USA) [2], and first-pass perfusion imaging (base, mid, and apex; 0.05 mmol/kg gadolinium) at rest and in response to 140 mcg/kg per minute adenosine stress for calculation of myocardial perfusion reserve index (MPRI) [6]. Image analysis for MPRI was performed manually using a DICOM viewer (OsiriX by Pixmeo, Bernex, Switzerland).

Angina was assessed before and after treatment using the Seattle Angina Questionnaire [7].

Individual data are presented wherever possible. Data comparing the treatment effect are presented as mean ± standard error. Linear regression was used to determine the relationship between diastolic function and angina frequency. Due to the small sample size, statistical analysis was not performed.

## Results

Patient characteristics are reported in Table 1. A total of five women enrolled, four completed the trial, while one was withdrawn by the investigators after developing dyspnea 1 week after treatment. Her symptoms resolved after cessation of the study medication.

**Fig. 1:**
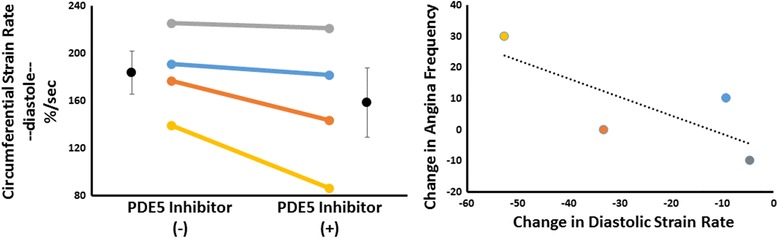
Phosphodiesterase 5 inhibition worsens diastolic function and symptoms of angina in women with ischemic syndrome. *Left*: Magnetic resonance tissue tagging was performed on two consecutive mid-short axis slices before and after treatment, and analyzed using commercially available software to assess the rate of circumferential strain in diastole. Individual data are shown, illustrating a reduction in all four patients who completed the trial. Group data (mean and standard error) are also reported (*black markers*). *Right*: Relationship between changes in self-reported angina frequency (Seattle Angina Questionnaire) and the change in diastolic circumferential strain rate. *PDE5* phosphodiesterase 5

The major novel finding of this investigation was that PDE5 inhibition reduced the rate of circumferential strain in diastole in all four women who completed the trial (that is, diastolic dysfunction; Fig. 1). This impairment could not be explained by: changes in heart rate, that is, 58 ± 6 beats per minute (bpm) versus 61 ± 6 bpm; contractility, that is, left ventricular (LV) ejection fraction 70 ± 1% versus 67 ± 1% and peak circumferential strain −20.7 ± 1.0% versus −20.2 ± 1.4%; or preload, that is, LV end-diastolic volume 108 ± 8 ml versus 109 ± 3 ml and left atrial volume 56 ± 10 ml versus 61 ± 11 ml; and was not associated with a change in MPRI 2.32 ± 0.10 versus 2.39 ± 0.06 (pre versus post).

The frequency of angina also tended to increase with treatment, with the greatest increase occurring in the patient with the greatest impairment in diastolic strain (Fig. 1).

## Discussion

In contrast to our hypothesis, PDE5 inhibition appears to worsen left ventricular relaxation in women with angina but no evidence of obstructive CAD. That one patient developed dyspnea while on the drug is consistent with the diastolic changes observed in the other four patients, and raises concern about the safety of PDE5 inhibition in this population. Our results add to a growing body of literature questioning the safety [8] and efficacy [9] of PDE5 inhibition to treat heart disease, currently an off-label indication (other than pulmonary hypertension).

The mechanism responsible for the reduction in diastolic function remains unknown. We did not observe frank hemodynamic changes, arguing against extrinsic factors. It is therefore interesting to speculate that adverse cellular signaling may have played a role, especially since the two patients with the greatest impairment/symptoms (P4 and P5) were treated with sildenafil, which is far less selective for PDE5 and also inhibits PDE1, which hydrolyzes cyclic adenosine monophosphate (cAMP) as well as cGMP.

This study is not without limitation however. For example, the small sample size and lack of a control group limits the broad application and statistical verification of these results. Moreover, participants were not randomized, nor were they blinded to the treatment. Despite these limitations, however, the uniform nature of our results warrants serious consideration and future investigation. In particular, a randomized placebo-controlled trial in a larger study sample is needed to fully elucidate the therapeutic efficacy of PDE5 inhibitors in CMD.

## Conclusion

Taken together, these data question the efficacy of phosphodiesterase type 5 inhibition to treat women with ischemic heart disease, and highlight the need for further investigation.

## Abbreviations

bpm: Beats per minute
CAD: Coronary artery disease
cGMP: Cyclic guanosine monophosphate
CMD: Coronary microvascular dysfunction
LV: Left ventricular
MPRI: Myocardial perfusion reserve index
MRI: Magnetic resonance imaging
NHLBI: National Heart, Lung, and Blood Institute
PDE: Phosphodiesterase
WISE: Women’s Ischemia Syndrome Evaluation
